# Prognostic Value of Circulating Inflammatory Cells in Patients with Stable and Acute Coronary Artery Disease

**DOI:** 10.3389/fcvm.2017.00044

**Published:** 2017-07-14

**Authors:** John A. L. Meeuwsen, Marian Wesseling, Imo E. Hoefer, Saskia C. A. de Jager

**Affiliations:** ^1^Laboratory for Experimental Cardiology, University Medical Center Utrecht, Utrecht, Netherlands; ^2^Laboratory for Clinical Chemistry and Hematology, University Medical Center Utrecht, Utrecht, Netherlands; ^3^Laboratory of Translational Immunology, University Medical Center Utrecht, Utrecht, Netherlands

**Keywords:** inflammatory cells, coronary artery disease, biomarkers, acute coronary syndromes, stable coronary artery disease, circulating cells, follow-up

## Abstract

Atherosclerosis is a lipid driven chronic inflammatory disease underlying the majority of ischemic events such as myocardial infarction or stroke. Clinical management of ischemic events has improved considerably in the past decades. Accordingly, survival rates have increased. Nevertheless, 12% of patients die within 6 months after the initial event. To improve secondary prevention, appropriate risk prediction is key. However, up to date, there is no clinically available routine marker to identify patients at high risk for recurrent cardiovascular events. Due to the central role of inflammation in atherosclerotic lesion progression and destabilization, many studies have focused on the role of circulating inflammatory cells in these processes. This review summarizes the current evidence on the potential of circulating inflammatory cells as biomarkers for recurrent adverse manifestations in acute coronary syndrome and stable coronary artery disease patients.

## Introduction

Cardiovascular disease (CVD) remains one of the most important causes of death worldwide. It represents a major challenge in healthcare and it is estimated that ~17.5 million people die from CVD annually. Although treatment and interventional options have significantly improved survival rates, consequently the incidence of chronic CVD is expected to increase in the next decades (1). Currently, approximately 90% of patients that encounter a first myocardial infarction (MI) survive (2). However, the risk of secondary cardiovascular manifestations is high. Roughly 5–15% of patients die during hospitalization or within 30 days after MI (3–5) and an additional 5% of patients die within 6 months upon hospital discharge (6). The risk of re-infarction is most profound within the first year, occurring in approximately 17% of patients (7), and the risk for recurrent adverse events further increases over time (8).

Atherosclerosis is the culprit pathology driving coronary artery disease (CAD) and is characterized by the presence of lipid and inflammatory cells during initiation, progression and destabilization of an atherosclerotic plaque (9–11). Monocytes and macrophages are well known for their role in plaque growth. Monocytes transmigrate through the disrupted endothelium and infiltrate the intimal area, where they mature into macrophages. By phagocytosis of oxidized LDL, they develop into foam cells which form an integral part of atherosclerotic plaques (12). Next to monocytes, neutrophils, mast cells, and (activated) lymphocytes massively accumulate in rupture-prone regions of the atherosclerotic plaques, suggesting an important role in plaque destabilization (13–15). As the atherosclerotic lesion progresses, necrotic cell death occurs, a process that promotes further lesion progression as it contributes to the inflammatory response and enlargement of the necrotic core, which cumulatively can result in plaque rupture (16–18). Whether the process of necrosis in the plaque directly alters the circulating inflammatory cells profile has, to the best of our knowledge, not been established. Acute coronary syndromes (ACSs) can result in obstructed blood flow, with myocardial necrosis and inflammation as a consequence. In response, the circulating cell profile is altered and inflammatory cells are actively requited to the damaged myocardial tissue (16).

Given their prominent role in CVD, the sheer number of circulating inflammatory cells and their distribution into subpopulations may have predictive value for recurrent cardiovascular manifestations. As such, they can be used to improve risk stratification on top of existing prediction models [i.e., Global Registry of Acute Coronary Events (GRACE) risk score or the Thrombolysis in Myocardial Infarction (TIMI) risk score for ACS patients] (19). Identifying patients at high risk may help to prevent the occurrence of re-infarction or death.

Many studies have studied circulating inflammatory cells as markers of recurrent adverse manifestations after MI. Therefore, we aimed to provide an overview on the value of circulating inflammatory cells as markers for secondary events and mortality in stable CAD and ACS patients.

## The Prognostic Value of Circulating Inflammatory Cells in CAD

We have included studies that prospectively examine the predictive value of total white blood cell (WBC) count and WBC subtype count or ratios in patients with CAD or ACS specifically. The primary endpoints include major adverse cardiovascular events (MACE) or mortality.

## White Blood Cells

It is generally acknowledged that a high WBC count roughly reflects an activated inflammatory status of an individual. It is well known that people with inflammatory or autoimmune disorders have an increased risk of CVD (20–22), which is most likely the consequence of inflammatory responses negatively affecting plaque stability (23). Therefore, WBC count is well studied during the past decades in CAD patients. Although total WBC was not associated with plaque progression in one study (24), it has been associated with presence, severity, and extent of coronary atherosclerosis in another study (25), as well as with in-stent restenosis in PCI patients (26). The prognostic value of total WBC count in CAD patients is not consistent (Tables 1 and 2). On one hand, an elevated WBC count (>8.2 × 10e9/L) has been independently associated with risk of adverse events and mortality (2.2-fold increase on average) (27–36), but on the other hand, others show no independent predictive value of total WBC count in patients with CAD (37–43). These different findings might be explained by the fact that WBC count independently prognosticated outcome primarily in studies conducted on PCI patients, containing a mixed population of both acute and stable CAD (Tables 1 and 2). More specific prognostic information for risk of follow-up events or mortality might be derived from the WBC differential, since the different WBC subtypes have distinct roles in immune (dis)balance.

**Table 1:** Mortality predicted by total white blood cell (WBC) and WBC differential in coronary artery disease (CAD) patients categorized by follow-up time.

Study characteristics			WBC		Monocytes		Neutrophils		Lymphocytes		Neutrophil to lymphocyte ratio		Reference
						
Population	FU	Size (*n*)	Risk [95% CI]	*p*	Risk [95% CI]	*p*	Risk [95% CI]	*p*	Risk [95% CI]	*p*	Risk [95% CI]	*p*	
PCI	<1	309	1.2 [1.0–1.3]^b^	*									(27)
PCI	1–6	1,425	4.8 [1.3–16.8]	n.s.	1.7 [1.0–2.9]	n.s.	5.7 [1.6–19.6]	n.s.			5.6 [1.6–19.8]	**	(40)
PCI	>6–36	83	14.7 [2.7–80.7]	**									(29)
UAP/NSTEMI	>6–36	280	1.4 [0.6–2.9]	n.s.									(41)
CAD	>6–36	1,246	2.0 [1.3–3.1]	**									(36)
CAD	>6–36	422	5.3 [1.2–24.1]	n.s.	6.4 [1.4–28.8]	n.s.	5.5 [1.2–24.7]^c^	n.s.	4.2 [1.4–12.4]^a^	n.s.	8.1 [1.4–46.6]	*	(38)
PCI	>6–36	1,046									1.9 [1.3–3.0]	*	(44)
UAP/NSTEMI	>36	275	1.7 [1.1–2.5]	*									(34)
PCI	>36	1,425	1.7 [1.0–2.7]	n.s.	2.0 [1.2–3.4]	*	2.0 [1.2–3.3]	n.s.	0.6 [0.4–0.9]	n.s.	3.0 [1.8–5.0]	***	(40)

*The risk of mortality in CAD patients categorized by follow-up time. The indicated risk can be a relative risk, odds ratio, or hazard rate. Unless stated otherwise, the indicated risk regards the risk of patients in the group with the highest cell count compared to patients with low cell counts. FU indicates follow-up time in months*.

*^a^Risk regarding cell percentage*.

*^b^Risk increase per 1 × 10e9 cells per liter of blood*.

*^c^Risk was also not significant for neutrophil percentage*.

Significance in multivariate analyses is indicated by *p < 0.05; **p < 0.01; ***p < 0.001; n.s. indicates not significant.

**Table 2:** Cardiovascular events predicted by total white blood cell (WBC) and WBC differential in CAD patients categorized by follow-up time.

Study characteristics			WBC		Monocytes		Neutrophils		Lymphocytes		Neutrophil to lymphocyte ratio (NLR)		Reference
						
Population	FU	Size (*n*)	Risk [95% CI]	*p*	Risk [95% CI]	*p*	Risk [95% CI]	*p*	Risk [95% CI]	*p*	Risk [95% CI]	*p*	
AP	1–6	1,125	2.8 [1.9–4.3]	***			2.5 [1.6–3.7]	***			1.7 [1.1–2.5]	**	(33)
CAD	>6–36	389	1.3 [0.6–2.8]	n.s.	1.6 [1.1–2.5]	*	1.3 [0.7–2.3]	n.s.	0.8 [0.5–1.4]	n.s.			(42)
IS/MI/PAD	>6–36	18,558	1.4 [1.3–1.6]	***	1.2 [1.1–1.4]	**	1.5 [1.3–1.7]	***	1.0 [0.9–1.2]	n.s.			(30)
AP	>6–36	1,125	2.5 [1.7–3.7]	***			2.3 [1.6–3.4]	***			1.6 [1.1–2.3]	*	(33)
PCI	>6–36	140	34.0 [4.1–281]	**					37.5 [4.5–311]^a^^,^^d^	***			(28)
PCI	>6–36	83	10.9 [2.4–49.7]	**									(29)
PCI SVG	>6–36	530	1.2 [1.1–1.3]^c^	***									(32)
UAP/NSTEMI	>6–36	280	1.3 [0.7–2.3]	n.s.									(41)
ACP neg	>6–36	975	NR	n.s.	NR	n.s.	NR	n.s.	2.5 [1.3–4.8]^d^	**			(39)
Stable CAD	>6–36	422	2.1 [1.0–4.4]	n.s.			1.8 [0.9–3.8]^e^	n.s.			NR	n.s.	(38)
Angiography	>6–36	951			3.0 [1.3–6.9]^g^	**							(45)
CAD	>6–36	263			4.0 [1.3–12.1]^b^	*							(46)
Stable CAD	>6–36^f^	141							1.7 [0.8–3.4]^a^	n.s.			(47)
UA	>6–36	120							3.0 [1.1–8.3]^h^	*			(48)
Angiography	>6–36	3,005									1.6 [1.1–2.2]	*	(49)
(U)AP	>36	3,227	1.4 [NR]	*	1.3 [NR]	*	1.8 [NR]	***	0.5 [NR]	***	2.2 [NR]	***	(31)
CHD	>36	942	0.9 [0.9–1.0]	n.s.							1.1 [1.1–1.2]^c^	***	(43)
CHD	>36	4,535	1.4 [1.0–1.9]	NR									(35)
Stable CAD	>36	2,370	1.4 [0.7–2.5]	n.s.							1.7 [1.3–2.2]	***	(37)
Stable CAD	>36	141							2.3 [1.3–3.8]^a^	NR			(47)
PCI	>36	798									2.3 [1.3–4.3]	**	(50)

*The risk of major adverse cardiovascular events (MACE) in CAD patients categorized by follow-up time. The indicated risk can be a relative risk, odds ratio, or hazard rate. Unless stated otherwise, the indicated risk regards the risk of patients in the group with the highest cell count compared to patients with low cell counts. FU indicates follow-up time in months*.

*^a^Risk regarding cell percentage*.

*^b^Risk regarding >15% urokinase-type plasminogen activator receptor-positive monocytes*.

*^c^Risk increase per 1 × 10e9 cells per liter of blood or increase of 1 NLR*.

*^d^Lowest tertile or quartile compared to highest*.

*^e^Besides neutrophil count, the neutrophil percentage was also not associated with MACE risk*.

*^f^Risk score was also not related for 3 years of follow-up*.

*^g^CD14^+^CD16^++^ monocytes; total monocyte count or other subsets were not associated with MACE*.

*^h^Risk regarding CD4^+^CD28^null^ T cell frequency >4%*.

The level of significance in multivariate analyses is indicated by *p < 0.05; **p < 0.01; ***p < 0.001; n.s. indicates not significant.

## Monocytes

Plaque macrophages derive from infiltrating monocytes and are well known for their role in atherosclerotic initiation and development (24, 51, 52). Distinct monocyte subsets exist and differ in function (53). Classical CD14^++^CD16^−^ monocytes are predominantly phagocytic, non-classical CD14^+^CD16^++^ monocytes display mainly inflammatory characteristics, and a small transitional subset of intermediate CD14^+^CD16^+^ monocytes displays both phagocytic and inflammatory function (54). In a few studies exploring the prognostic power of total monocytes, no evidence for monocytes as independent predictors of recurrent cardiovascular events could be established (38, 39). However, others report that elevated monocyte count is a significant predictor for secondary cardiovascular manifestations and mortality increasing the average risk by 50% (30, 31, 40, 42, 45). This has been shown to be primarily associated with the CD14^+^CD16^++^ intermediate monocyte subtype (45) and to be dependent on follow-up time (40) (Tables 1 and 2). Another study investigating the role of monocyte activation showed that patients with high percentages of urokinase-type plasminogen activator receptor-positive monocytes, indicative of increased monocyte infiltration and activation, had increased risk of recurrent cardiovascular events (46).

## Neutrophils

Both under homeostatic and acute inflammatory conditions, neutrophils are the major component of the total WBC count (~60–70%). Although rarely found in human atherosclerotic plaques, experimental atherosclerosis mouse studies have shown a role for neutrophils in the initiation of atherosclerosis (55). Elevated neutrophil levels are associated with rupture prone plaques (56, 57) and in-stent restenosis after PCI (26). However, there is conflicting evidence about the prognostic value of neutrophils in stable CAD patients. Some reports show that elevated neutrophil count (>6 G/L) is associated with an approximately doubled risk of mortality or MACE (30, 31, 33). Others could not establish this prognostic value of neutrophils (38–40, 42) (Tables 1 and 2). The studies that showed an independent prognostic value of neutrophils were more likely to include PCI and AP patients instead of CAD patients, and to have MACE as endpoint rather than mortality. This suggests that, also in the setting of CVD, neutrophils are mostly related to an acute inflammatory reaction upon cardiac injury rather than the chronic inflammatory response in plaque development.

## Lymphocytes

The role of lymphocytes in CVD has been extensively investigated in experimental atherosclerosis models. A plethora of different B- and T-cell subtypes with both atherogenic or atheroprotective roles has been reported (reviewed in Ref. 58). However, in the human setting, the evidence for a lymphocyte specific role in the presentation of atherosclerosis is limited and the outcome remains inconclusive. The majority of studies showed that high lymphocyte counts were not independently associated with plaque progression, in-stent restenosis, CVD events, or mortality (24, 26, 30, 38, 40), while there is only one study that reports on high levels of lymphocytes being protective for future events (31) (Tables 1 and 2). Another group observed a role for a specific T lymphocyte subset, as the increase of CD4^+^CD28^null^ T cells was associated with increased rate of CVD events (48) (Table 2). These CD4^+^CD28^null^ cells have profound effector functions and are considered to be pro-atherogenic as their levels are high in unstable, but not in stable plaques (59). When assessing lymphocytes as a percentage of the total WBC count, high lymphocyte percentages were protective of 1-year MACE in PCI patients (28). By contrast, high lymphocyte percentages showed to be detrimental in stable CAD patients for long term follow-up (3 and 13.2 years) only (47). It can be debated on whether using the percentage of WBCs is the best parameter as the relative contribution of inflammatory cell subtypes may be similar in patients with or without follow-up events. However, due to an increase in total WBC counts, the total amount of cell subtypes may very well differ between patients.

## Neutrophil to Lymphocyte Ratio (NLR)

The NLR is an emerging biomarker that may better reflect the immune status of an individual as compared to a single inflammatory cell subtype. In a recent study using multidetector computed tomography, NLR was shown to be associated with severity and plaque morphology in CAD patients (60). The NLR prognosticates outcome consistently in the majority of the studies performed in stable CAD patients (Tables 1 and 2). Risk of MACE or mortality increased approximately by 150% in patients with elevated NLR (average cut-off >2.5) (31, 33, 37, 38, 40, 43, 44, 49, 50). The exact underlying mechanisms of NLR in relation to recurrent events in CAD patients are poorly understood. Low lymphocyte counts might result from increased cortisol levels that induce apoptosis specifically in lymphocytes (and eosinophils), but increase total WBC count (61). A rise in neutrophil counts is often accompanied with increased neutrophil activity, thereby leading to the release of proteolytic enzymes, for example, myeloperoxidase, which can induce tissue injury (62). In addition, vascular obstruction as a consequence of neutrophil aggregates, a process also involving platelets and endothelial cells, increases the risk of recurrent events (63).

## The Prognostic Value of Circulating Inflammatory Cells in ACSs

In patients with ACS, the rate of recurrent cardiovascular events and mortality is higher as compared to stable CAD patients. Because of the increased event rate and the differences in plaque composition (64, 65), the prognosis in these patients is likely to be different as well. Therefore, we consider the prognostic value of circulating cells in ACS patients separately in this chapter.

## White Blood Cells

Similar to more general CAD populations, also in specific ACS populations contrasting results are reported for the prognostic value of WBCs. Several reports show that increased leukocyte count (on average >12 × 10e9/L) predicted increased MACE and mortality risk independently (approximately 2.5 times risk increase) (3, 66–76). However, these findings were not corroborated by others (77–84) (Tables 3 and 4). In general, WBC count was a better predictor in patients with short follow-up than long follow-up. In addition, the studies which showed a prognostic value for the WBC count had a larger sample size, which might explain the different observations in prognostic value of WBCs. How the different WBC subtypes may relate to the risk for MACE or mortality during follow-up will be discussed below.

**Table 3:** Mortality risk predicted by total white blood cell (WBC) and WBC differential in acute coronary syndrome (ACS) patients categorized by follow-up time.

Study characteristics			WBC		Monocytes		Neutrophils		Lymphocytes		Neutrophil to lymphocyte ratio		Reference
						
Population	FU	Size (*n*)	Risk [95% CI]	*p*	Risk [95% CI]	*p*	Risk [95% CI]	*p*	Risk [95% CI]	*p*	Risk [95% CI]	*p*	
AMI	<1	153,213	2.3 [2.2–2.5]	***									(3)
AMI	<1	751	2.3 [1.2–4.5]	*									(66)
AMI	<1	1,016	3.7 [1.6–8.7]	**									(67)
AMI	<1	115,273	2.7 [2.5–2.9]	NR									(68)
AMI	<1	2,863	1.6 [1.1–2.5]	**									(76)
STEMI	<1	305					4.6 [1.5–14.4]^a^	*					(85)
STEMI	<1	404					2.9 [1.0–8.4]	*					(86)
STEMI + pPCI	<1	304									1.1 [1.0–1.2]	n.s.	(87)
STEMI	<1	522									3.8 [1.7–8.3]	**	(88)
ACS	<1	2,833									2.0 [1.2–3.6]	*	(89)
STEMI	<1	636									2.4 [1.3–4.4]	n.s.	(90)
STEMI + PCI	<1	538									15.8 [1.6–154]	*	(91)
ACS	1–6	2,833									3.9 [3.2–8.1]	***	(89)
STEMI	>6–36	470	2.5 [1.3–4.9]	NR	1.8 [1.0–3.4]	NR	2.2 [1.2–4.3]	NR	0.4 [0.2–1.0]	NR	4.2 [1.7–10.2]	**	(74)
AMI	>6–36	1,016	2.9 [1.2–7.1]	*									(67)
ACS + PCI	>6–36	4,329	1.1 [1.0–1.1]^b^	***									(70)
pPCI	>6–36	958	1.1 [1.0–1.2]^b^^,^^d^	NR									(71)
AMI	>6–36	447	NR	n.s.									(81)
NSTE ACS	>6–36	1,315	1.5 [1.1–2.0]	*									(72)
MI	>6–36	2,047					2.3 [1.8–2.8]	***					(86)
STEMI + pPCI	>6–36	304									1.1 [1.1–1.2]	n.s.	(87)
STEMI + pPCI	>6–36	210									2.7 [1.0–7.2]	*	(92)
STEMI + PCI	>6–36	325									3.1 [1.1–8.6]	*	(93)
NSTEMI	>36	619	1.0 [0.9–1.2]	n.s.	0.1 [0.0–1.1]	n.s.	1.0 [0.9–1.2]	n.s.	0.6 [0.2–1.7]	n.s.	1.1 [1.0–1.1]	**	(83)
STEMI	>36	458	1.6 [1.1–2.3]	**									(73)
NSTE-ACS	>36	476	2.0 [1.4–2.7]	***									(73)
AMI	>36	144	1.3 [NR]	n.s.									(78)
STEMI + PCI	>36	1,377							2.4 [1.3–4.7]^c^	***			(94)
STEMI + PCI	>36	538									2.2 [1.0–4.8]	*	(91)

*The risk of mortality in ACS patients is categorized by follow-up time. The risk can be relative risk, odds ratio or hazard rate. Unless otherwise stated, the indicated risk regards the risk of patients in the group with the highest cell count compared to patients with low cell counts. FU indicates follow-up time in months*.

*^a^Risk regarding cell percentage*.

*^b^Risk increase per 1 × 10e9 cells per liter of blood*.

*^c^Lowest tertile or quartile compared to highest*.

*^d^The presented risk score is not significant for both 1 and 2.6 years of follow-up*.

The level of significance in multivariate analyses is indicated by *p < 0.05; **p < 0.01; ***p < 0.001; n.s. indicates not significant.

**Table 4:** Cardiovascular events predicted by total white blood cell (WBC) and WBC differential in acute coronary syndrome (ACS) patients categorized by follow-up time.

Study characteristics			WBC		Monocytes		Neutrophils		Lymphocytes		Neutrophil to lymphocyte ratio		Reference
						
Population	FU	Size (*n*)	Risk [95% CI]	*p*	Risk [95% CI]	*p*	Risk [95% CI]	*p*	Risk [95% CI]	*p*	Risk [95% CI]	*p*	
AMI	<1	1,016	2.0 [1.1–3.6]	*									(67)
pPCI	<1	80	1.3 [1.0–1.5]^b^	*									(69)
ACS/nSTEMI	<1	352	2.5 [1.4–5.7]	n.s.									(80)
AMI	<1	177	1.0 [1.0–1.1]	n.s.									(82)
nSTE ACS	<1	160					6.5 [1.6–27.2]	*					(95)
AMI + pPCI	<1	440									1.3 [1.2–1.5]	*	(96)
STEMI + pPCI	<1	304									1.1 [1.0–1.6]	n.s.	(87)
STEMI	<1	682									1.2 [1.0–1.3]	***	(97)
STEMI	<1	101									3.6 [1.2–10.7]	*	(98)
STEMI + pPCI	<1	170									1.2 [1.0–1.3]	*	(99)
ACS	>6–36^c^	2,661	0.9 [0.9–1.0]	n.s.			1.0 [0.8–1.0]	n.s.	0.9 [0.8–1.0]	n.s.			(84)
STEMI + PCI	>6–36	331	2.4 [1.4–4.3]	**							3.8 [2.2–6.7]	***	(75)
MI	>6–36	107	NR	n.s.									(78)
MI	>6–36	64	0.4 [0.2–2.1]	n.s.									(79)
STEMI	>6–36	100			3.6 [1.2–10.8]^d^	*							(100)
ACS	>6–36	166							1.2 [1.1–1.3]^a^	**			(101)
STEMI + pPCI	>6–36	304									1.1 [1.1–1.2]	n.s.	(87)
STEMI + pPCI	>6–36	326									3.8 [1.1–12.6]	*	(102)
STEMI	>36	1,287					1.3 [1.2–1.3]	***					(103)
STEMI	>36	682									1.3 [1.1–1.3]	***	(97)

*The risk of major adverse cardiovascular events (MACE) in ACS patients is ordered by follow-up time. The risk can be relative risk, odds ratio or hazard rate. Unless otherwise stated, the indicated risk regards patients in the group with the highest cell count compared to patients with low cell counts. FU indicates follow-up time in months*.

*^a^Risk increase regarding CD28^null^ CD4 T cell percentage*.

*^b^Risk increase per 1 × 10e9 cells per liter of blood*.

*^c^The presented risk is for all cell types also not significant for in-hospital MACE*.

*^d^Total monocytes, CD14^++^CD16^−^ and CD14^++^CD16^+^, but not CD14^+^CD16^++^ monocytes, were predictive when measured at post MI day 2*.

The level of significance in multivariate analyses is indicated by *p < 0.05; **p < 0.01; ***p < 0.001; n.s. indicates not significant; NR indicates not reported.

## Monocytes

Although monocytes play a central role in atherosclerosis, their role in risk prediction has been scarcely investigated in ACS patients (Tables 3 and 4). In one study, the three monocyte subsets were investigated over time after MI, and monocyte numbers peaked at day 2 post-MI (100). Total, CD14^++^CD16^+^ classical monocytes and CD14^++^CD16^−^ non-classical monocytes, but not CD14^+^CD16^++^ intermediate monocytes, were predictive for MACE when assessed 2 days post-MI. Of five different measurements over time in this study, CD14^++^CD16^+^ classical monocytes were most consistently (four out of five) associated with recurrent CVD events (100). In addition, the classical CD14^++^CD16^+^ monocyte was significantly associated with carotid atherosclerosis and intraplaque neovascularization (104). In two other studies, the total monocyte count did not independently prognosticate mortality (74, 83). Thus, there is limited and inconclusive evidence for a prognostic value of circulating monocytes for recurrent CVD events and mortality, despite the fact that monocytes are important in plaque development and rupture. The lack of a clear association may have different explanations. It can be due to the fact that mainly total monocyte numbers are used, while it has been clearly established that different monocyte subtypes exist (54). Indeed, in line with the findings in stable CAD patients, a significant association with recurrent cardiovascular events was observed in specific monocyte subsets (45, 46, 100). On the other hand, the circulating monocyte pool may simply not reflect the macrophage pool in the plaque. Indeed, a study by Robbins and colleagues has established that plaque macrophages in a murine atherosclerosis model mostly derive from proliferation of the existing macrophage pool rather than recruitment of new monocytes (105). Furthermore, besides technical issues such as different gating strategies or isolation methods, monocyte plasticity could also explain why the circulating monocytes do not *per se* mirror plaque macrophages. Indeed, experimental evidence indicates that plaque macrophages can switch phenotype over time in advanced atherosclerotic lesions (106, 107).

## Neutrophils

As described in CAD patients, conflicting reports were published about the prognostic value of neutrophil counts. In ACS patients, the results are also not uniform, though more positive. Two studies were unable to establish an independent predictive value for neutrophil counts (83, 84). However, in the majority of the studies assessing the prognostic value of neutrophils, their counts significantly predicted short term, i.e., <30 days, (average OR 5) (85, 86, 95) and long term, i.e., 3 years, (average HR 1.75) (74, 103, 108) secondary cardiovascular events and mortality (Tables 3 and 4). Although different studies have described that elevated neutrophil counts associate with worse outcome, it is surprising that there is no consistent evidence. Elevated neutrophil counts have been associated with endothelial disruption as a consequence of released reactive oxygen species and MPO (62). In addition, neutrophils can induce vascular plugging, thereby extending infarct size (63). The relative neutrophil count (as percentage of total WBC) might add prognostic value, but only few studies have examined their predictive power. However, the NLR described later in this review has been studied extensively.

## Lymphocytes

There is limited evidence for a role of total lymphocytes in risk prediction of ACS patients. Total lymphocyte counts were not independently associated with follow-up events and mortality (74, 83, 84) except for one study, where low lymphocyte counts were predictive of mortality (94) (Tables 3 and 4). In addition, as for CAD patients, an increase of pro-atherogenic CD4^+^CD28^null^ T cells was associated with increased rate of recurrent CVD events (101) (Table 4). The information on the prognostic value of lymphocytes and specific lymphocyte subtypes is limited. Considering that both T and B cell subsets can have distinct pro- or anti-atherogenic or inflammatory characteristics (58, 109), the prognostic value of these specific subtypes could hold more value compared to the complete lymphocyte count. With advances made in multicolor flow cytometry the last decade (110), this research area deserves more attention in future biomarker studies.

## Neutrophil to Lymphocyte Ratio

Although the number of studies describing neutrophil or lymphocyte counts alone is limited and inconclusive, the NLR has been well studied during the past years (Tables 3 and 4). Similar to stable CAD patients, elevated NLR prognosticates adverse outcome in ACS patients as shown by several studies both at short term, i.e., <30 days (88, 89, 91, 97–99), and long term, i.e., >6 months, of follow-up (74, 75, 83, 89, 91–93, 97, 102) with an average fourfold increased risk. Only in two studies, the NLR was not independently associated with MACE and CVD mortality (87, 90). Apparently, the measurement combining neutrophil and lymphocyte counts adds significantly to the prognostic power of neutrophils or lymphocytes alone.

## Summary and Future Perspectives

Adequate risk assessment for patients with stable and unstable CAD is crucial to prevent recurrent events. Total circulating WBCs and WBC subtypes are strongly associated with risk of recurrent adverse events independently of many risk factors, both during hospitalization and up to 10 years of follow-up. Overall, elevated NLR appears to be most consistently associated with adverse outcomes in CAD and ACS patients. In comparison to the WBC count, four out of eight studies found that the prognostic value of NLR was superior (37, 38, 40, 83). The remaining four studies showed comparable prognostic power for NLR and WBC count (31, 33, 74, 75). Moreover, although limited, there is evidence that the NLR adds significantly to the prognosis assessed by existing prediction models such as the GRACE (83, 89, 98) and TIMI (102) risk scores. Despite the fact that it should be thoroughly established that NLR has added value in existing risk prediction models like the TIMI and GRACE risk score (111, 112), clinical implementation of NLR can be reached relatively quickly. The NLR is an inexpensive marker and can easily be calculated from the WBC differentiation that is routinely analyzed in every hospital. Of note, several factors may influence the prognostic value of NLR. For instance, the NLR is higher in women below 50 years of age compared to age matched man, while in postmenopausal women, NLR is lower compared to age matched men (113). Besides, ethnical differences may also influence the WBC profile. The NLR is lower in African-Americans as compared to other ethnicities, including Caucasian, Asian, and Hispanic (114–116). Therefore, a tailored cut-off value of the NLR for age, sex and ethnicity might lead to a more accurate risk prediction.

Caution should be taken regarding the limitations of circulating cells as biomarker. Numbers of circulating inflammatory cells, or their subtypes, do not necessarily reflect the amount or phenotype of these cells in the plaque (45, 46, 100, 105). Nevertheless, implementation of the NLR has a great potential to identify high risk CAD and ACS patients, thereby offering opportunities for intervention and prevention of recurrent cardiovascular events.

## Author Contributions

JM and MW designed and drafted the manuscript; IH and SJ designed and revised the manuscript.

## Conflict of Interest Statement

The authors declare that the research was conducted in the absence of any commercial or financial relationships that could be construed as a potential conflict of interest.
